# Investigating the relationship between subjective perception and unconscious feature integration

**DOI:** 10.1167/jov.24.12.1

**Published:** 2024-11-04

**Authors:** Lukas Vogelsang, Maëlan Q. Menétrey, Leila Drissi-Daoudi, Michael H. Herzog

**Affiliations:** 1 Laboratory of Psychophysics, École Polytechnique Fédérale de Lausanne (EPFL), Lausanne, Switzerland

**Keywords:** temporal integration, unconscious processing, sequential metacontrast paradigm, subjective perception, perceptual grouping

## Abstract

Visual features need to be temporally integrated to detect motion signals and solve the many ill-posed problems of vision. It has previously been shown that such integration occurs in windows of unconscious processing of up to 450 milliseconds. However, whether features are integrated should be governed by perceptually meaningful mechanisms. Here, we expand on previous findings suggesting that subjective perception and integration may be linked. Specifically, different observers were found to group elements differently and to exhibit corresponding feature integration behavior. If the former were to influence the latter, perception would appear to not only be the outcome of integration but to potentially also be part of it. To test any such linkages more systematically, we here examined the role of one of the key perceptual grouping cues, color similarity, in the Sequential Metacontrast Paradigm (SQM). In the SQM, participants are presented with two streams of lines that are expanding from the center outwards. If several lines in the attended motion stream are offset, offsets integrate unconsciously and mandatorily for periods of up to 450 milliseconds. Across three experiments, we presented lines of varied colors. Our results reveal that individuals who perceive differently colored lines as “popping out” from the motion stream do not exhibit mandatory integration but that individuals who perceive such lines as part of an integrated motion stream do show offset integration behavior across the entire stream. These results attest to the proposed linkage between subjective perception and integration behavior in the SQM.

## Introduction

Visual features need to be temporally integrated to process motion signals and solve the many ill-posed problems of vision. Consider, for instance, a car traveling at night, with its exterior reflecting light from nearby street lamps and its movement being occasionally obscured by other cars and objects. In such scenario, it is difficult to accurately estimate specific features of the car using only the activity of single photoreceptors at one moment in time. A more effective approach would be to average the activity of photoreceptors over the entire movement trajectory of the car. We have previously shown that, indeed, such integration takes place in long-lasting windows of unconscious processing that precede conscious awareness (Drissi-Daoudi, Doerig, & Herzog, 2019; Herzog, Drissi-Daoudi, & Doerig, 2020; Herzog, Kammer, & Scharnowski, 2016; Otto, Ögmen, & Herzog, 2006).

However, not all features in a visual scene should be integrated together. For instance, the motion signals of two cars driving in different directions should be processed separately, as should their colors. Drissi-Daoudi, Öğmen, and Herzog (2021) proposed that a complex grouping stage precedes temporal integration. They demonstrated that only in conditions where object integrity is preserved, features integrate along a motion trajectory. In one experiment, their data also pointed to inter-individual participants’ integration behavior being linked to whether they reported percepts of object integrity (Drissi-Daoudi et al., 2021).

Here, we followed up on these results and systematically tested whether different percepts, elicited by different perceptual groupings, are associated with different integration behaviors. To this end, we used the sequential metacontrast paradigm (or SQM), which is ideally suited for tackling this research question, as its methodology allows measuring mandatory, unconscious integration flexibly and systematically (Drissi-Daoudi et al., 2019; Drissi-Daoudi et al., 2020; Drissi-Daoudi et al., 2021; Menétrey, Herzog, & Pascucci, 2023; Otto et al., 2006; Otto, Ögmen, & Herzog, 2009; Otto, Ögmen, & Herzog, 2010; Plomp, Mercier, Otto, Blanke, & Herzog, 2009; Scharnowski et al., 2009; Vogelsang, Drissi-Daoudi, & Herzog, 2023; Vogelsang, Drissi-Daoudi, & Herzog, 2024).

**Figure 1.**
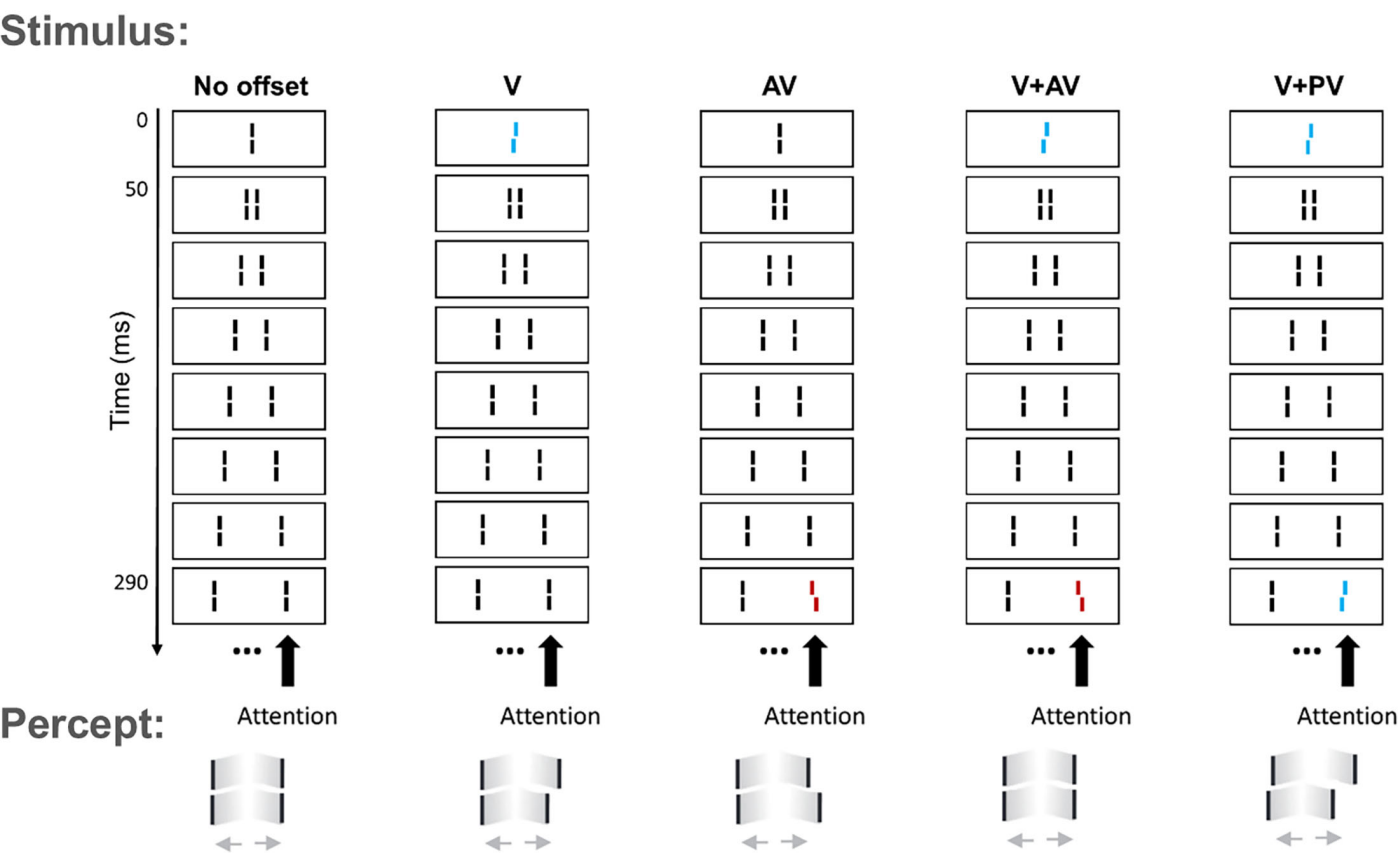
Illustration of the SQM. Stimulus presentation begins with the display of a central line, succeeded by sequential pairs of flanking lines, creating the percept of two motion streams that are diverging. Participants are required to focus on one of the two streams and are asked to indicate the direction in which they perceive an offset. If the line in the center is offset, the entire stream appears to be offset despite all other lines being straight. If two lines are offset, then the two offsets integrate if they are in the same stream, for up to 450 ms (Drissi-Daoudi et al., 2019). The result of this integration depends on the directions of the offsets: if they are in opposite directions, the offsets cancel each other out prior to becoming conscious and, thus, elicit the percept of a straight line. If the two offsets are presented in the same direction, offset discrimination increases. The colors in this depiction are for illustrative purposes only. The actual experiment utilized white lines on a black background. This illustration is adapted from Drissi-Daoudi et al. (2019).

In the SQM, as introduced in Otto et al. (2006), participants are presented with a central line, which is then succeeded by pairs of flanking lines. As illustrated in Figure 1, this creates the perception of two separate motion streams, which are diverging from the center. The central line is perceptually not visible because of metacontrast masking. However, if the central line is horizontally offset (termed “Vernier” offset or, more simply, “V”, where the line's lower part is shifted to either the left or the right, compared to the upper part), observers perceive the following lines as being offset in the same direction, despite them being actually straight. If one of the later lines has an offset as well, the two offsets integrate: if they are aligned in the same direction (referred to as “pro-Vernier” or “PV”), the ability to detect the offset is enhanced; if they are in opposite directions (known as “anti-Vernier” or “AV”), they counteract each other before reaching consciousness, resulting in the percept of a straight line. These effects hold even if the offsets are separated by up to 450 ms, as shown in Drissi-Daoudi et al. (2019). Furthermore, observers are not able to independently report the Vernier offsets separately. Hence, integration in the SQM is mandatory (Drissi-Daoudi et al., 2019).

**Figure 2.**
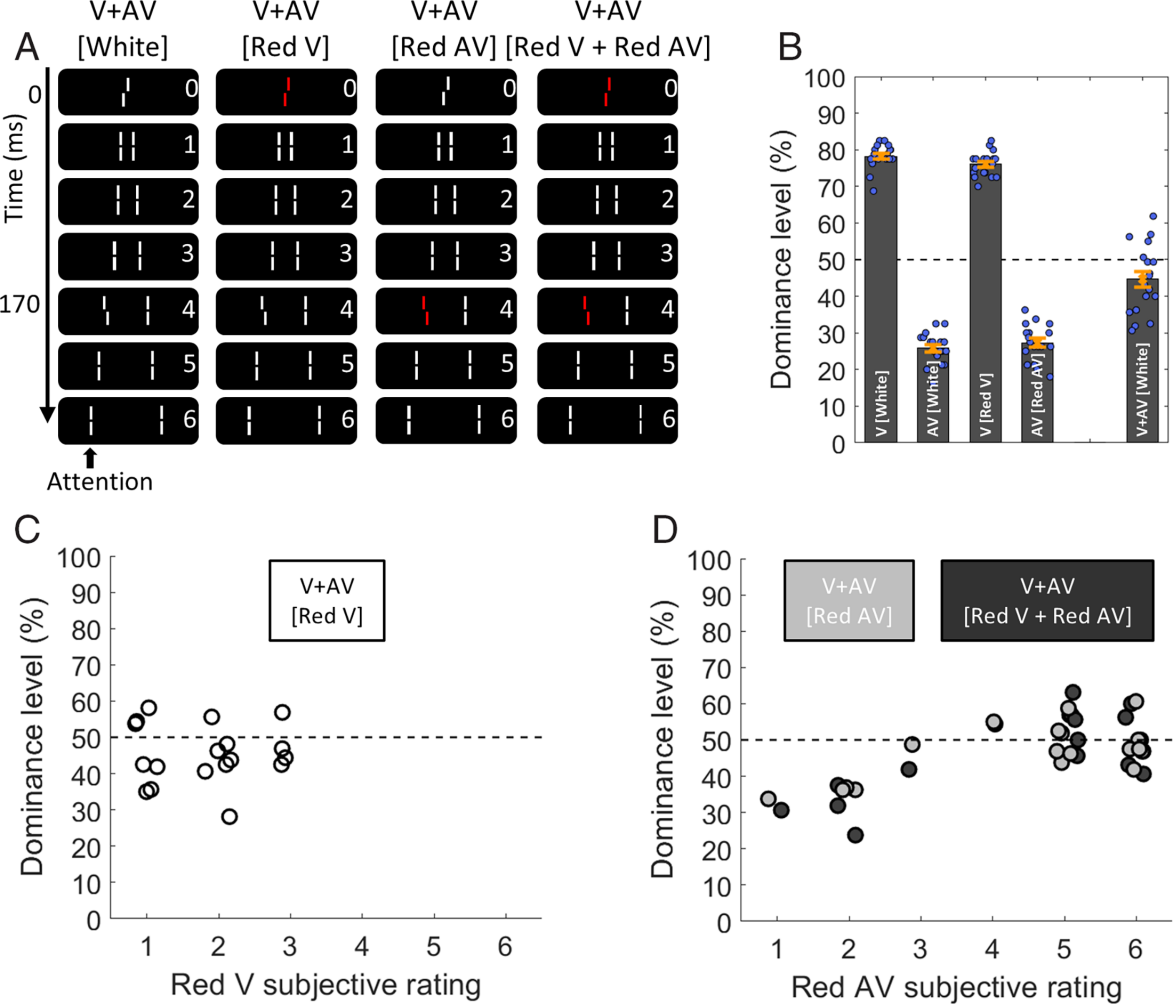
Results of experiment 1. (**A**) Visualization of the four different experimental conditions. (**B**) Central Vernier dominance levels. In the “White” condition, V and AV offsets integrate as expected (dominance around 50%). Error bars represent the standard error. (**C**) In the condition “Red V,” even though participants perceived the red element to be rather separated from the stream (low subjective ratings), V and AV offsets integrated (dominance around 50%). (**D**) In condition “Red AV,” many participants perceived the red AV as part of the stream (high subjective ratings), whereas some perceived it as separated from it (low subjective ratings). For the former, Vernier and anti-Vernier integrated (dominance around 50%), both in condition “Red AV” (gray) and “Red V + Red AV” (black). For the latter, V and AV offsets integrated less (dominance below 50%). Circles represent individual data points. Slight jitter along the x-axis was added for visualization; the real subjective ratings are integers.

Here, we asked whether different percepts are linked to different integration behaviors in the SQM. For instance, when some lines differ in terms of their color (see, Figure 2A), we hypothesize that integration depends on whether such line is perceived as part of the motion stream or as “popping out” from it. Across three experiments with the SQM, we present empirical tests of these predictions.

## Methods

### Participants

Participants were naïve to the experiment. They were recruited from École Polytechnique Fédérale de Lausanne as well as the University of Lausanne. All participants had normal or corrected-to-normal visual acuity, confirmed by scoring at least 1.0 on the Freiburg Visual Acuity test (Bach, 1996). The first experiment was completed by 18 individuals (aged between 19 and 30 years; including seven females), the second by eight participants (aged 19 to 28 years; three females), and the third by 18 participants (aged 18 to 24 years; nine females). Participants gave their consent after being fully informed about the study. After the study concluded, they received financial compensation. The research procedures complied with all ethical guidelines of the Declaration of Helsinki, except regarding preregistration. The study received approval from the ethics committee of canton Vaud in Switzerland (Commission cantonale d’éthique de la recherche sur l’être humain).

### Stimulus apparatus

Experimental stimuli were presented on a 24.5” BenQ XL2540 LCD monitor, featuring a resolution of 1920 × 1080 pixels and a 240 Hz refresh rate. MATLAB and Psychtoolbox (Brainard, 1997) were used for stimulus generation. Participants observed the visual stimuli from a distance of 2.5 meters from the screen, in a dimly lit room. Line stimuli were white (with a luminance of 100 cd/m²) or red (with a luminance of 20 cd/m²) and were presented on a background that was black. The monitor was set to 100 cd/m² for D65 white, and the RGB color space was linearized via a hardware gamma table.

### SQM stimuli

At the beginning of each trial, a fixation dot was presented for 0.5 seconds (for one second in experiment 1), followed by a blank screen for 0.5 seconds. The stimulus sequence then began, starting with the presentation of a central line comprising a lower and upper line segment of 26.5′ in length, separated vertically by 2.3′. Subsequently, matching pairs of flanking lines were displayed one pair at a time, moving progressively outward from the center. Whereas in experiments 1 and 2, flanking lines had the same vertical separation and length as the central line, in experiment 3, the line segment length increased by 1.6′ per pair of subsequent flanking lines, starting with 26.5′ at the center. For all experiments, individual lines measuring 1.2′ in width were set apart horizontally by 3.5′. Anti-aliasing was used for drawing the lines, to virtually increase the resolution of the offsets. Furthermore, the entire stimulus was jittered by ± 0.5 pixels on a trial-by-trial basis to avoid systematic anti-aliasing artefacts. Lines in the SQM were each presented for 21 ms. The inter-stimulus interval between the presentation of the central line and the presentation of the first set of flanking lines was 29 ms; the inter-stimulus interval between successive flanking line pairs was 21 ms. This arrangement of stimuli induced the percept of two diverging streams, moving from the center outward.

Participants were directed to attend covertly to the left motion stream (the right one in experiment 2). In the typical SQM stimulus sequence, the central line presented at the first frame had an offset (i.e., the lower segment of the line, relative to the upper line segment, was shifted either to the right or to the left). This offset line is termed “Vernier” or “V.” In some trials, a subsequent flanking line was also offset, but in the opposite direction, termed “anti-Vernier” or “AV.” If both offsets are presented together within the first 450 ms approximately, participants are not capable of reporting the individual offsets separately but, instead, perceive a single offset that is integrated.

The participants’ task was to discern and report, at the conclusion of the stimulus stream, whether they perceived the attended motion stream to be offset to the left or to the right. In addition, they were instructed to report the last perceived offset if more than one offset was seen along the motion stream. Following the display of the entire stimulus sequence, they had a three-second window to register their response via hand-held push buttons. The subsequent trial commenced after a brief interval (0.5 seconds in experiments 2 and 3, and one second in experiment 1). Although auditory feedback was given for incorrect responses as part of the initial calibration step (see section below), it was absent during the main experiment.

#### Experiment 1

In the attended motion stream, a central Vernier at frame 0 was followed by an anti-Vernier offset at frame 4. The SQM was presented with a total of six pairs of flanking lines. Four different main conditions were tested. In the “Classic” condition, all lines were presented in white. In the “Red V” condition, the central Vernier was red, but all other lines were white. In the “Red AV” condition, the anti-Vernier at frame 4 was red, but all other lines were white. Finally, in the “Red V + Red AV” condition, the central line and the fourth flanking line were red, but all other lines were white.

#### Experiment 2

Similar to experiment 1, a central Vernier at frame 0 was followed by an anti-Vernier at frame 4, with a total of six pairs of flanking lines being presented. Three different main conditions were tested. In the “Classic” condition, all lines were white. In the “Gradual Red” condition, the central line was white, the fourth line was red, but the red line was gradually faded in and out, by linearly interpolating the colors of the other lines (line 0: [255, 255, 255], line 1: [255, 191, 191], line 2: [255, 128, 128], line 3: [255, 64, 64], line 4: [255, 0, 0], line 5: [255, 64, 64], line 6: [255, 128, 128]). In the “Many Colors” condition, all lines in the attended motion stream had different colors (line 0: [255, 255, 255], line 1: [255, 255, 0], line 2: [255, 0, 0], line 3: [255, 0, 255], line 4: [0, 0, 255], line 5: [0, 255, 255], line 6: [0, 255, 0]). The given values refer to linear RGB value space.

#### Experiment 3

The SQM was presented with a central line and 9 flanking line pairs. The colors were alternating between each successive line. There were two different main conditions: a central Vernier at frame 0 presented together with an anti-Vernier at frame 3 (thus having different colors), and a central Vernier at frame 0 presented together with an anti-Vernier at frame 4 (thus having the same color). For half of the participants, the central Vernier was white (consequently, AV3 was red and AV4 was white); for the other half, the central Vernier was red (consequently, AV3 was white and AV4 was red).

### Offset size calibration

Before the main experiment, the offset sizes (i.e., the horizontal displacement between the lower and upper line segments) were calibrated for each relevant offset position and for each participant separately. This procedure was used to ensure that the offset directions of the different offset lines are comparably detectable. To do so, we presented sequences with only one offset line and utilized an adaptive parameter estimation by sequential testing procedure (Taylor & Creelman, 1967) to extract the offset size that is corresponding to 75% correct responses in reporting the offset direction.

In experiment 1, offset sizes were calibrated individually for frames 0 and 4, and separately for when the offset line was presented in white or red. In experiment 2, the calibration of offset sizes took place for frames 0 and 4, when lines were presented in white. For other colors, it was not recalibrated but later retested and corrected for in the analysis. In experiment 3, offset sizes were calibrated for frames 0, 3, and 4, for the specific colors that a given participant was presented with in the main experiment.

### Experimental procedure

For all main experiments, data was recorded in blocks of 80 trials each. If blocks were measured multiple times, performance was averaged across the respective repetitions as part of the subsequent analysis.

#### Experiment 1

Participants completed eight blocks—two blocks for each of the four main conditions introduced earlier: V + AV with one of the four different color conditions used: (i) white, (ii) colored V, (iii) colored AV, and (iv) colored V + colored AV. For the first four blocks, these conditions were displayed in random order. For blocks 5 through 8, the ordering was flipped. For instance, if the first four conditions appeared in the order (i) white, (ii) colored V, (iii) colored AV, (iv) colored V + colored AV, then the conditions were presented in the inverse order for the second half of blocks: (v) colored V + colored AV, (vi) colored AV, (vii) colored V, and (viii) white.

At the conclusion of the main experiment, participants were also asked to rate their percepts in the conditions “Red V” and “Red AV,” by providing a number between 1 and 6, with 1 representing “The red element appears to be completely separated from the motion stream” and 6 representing “The red element appears to be completely part of the motion stream.”

#### Experiment 2

Participants completed a total of 12 blocks. In the first six blocks, the V+AV conditions were measured for each of the three color conditions (“white,” “gradual red,” “many colors”) twice. Then, in blocks 7 through 9, the AV-only condition was measured for each of the three color conditions once. Finally, in blocks 10 through 12, the V-only condition was measured for each of the three color conditions once. The order in which the different color conditions were presented within each part was randomized.

Following the main part of experiment 2, further perceptual reports were collected from the participants. First, participants were shown 20 blocks of two identical trials each, depicting a non-offset SQM sequence with six pairs of flanking lines, in which the central line was either red or white. Participants were asked to verbally report (i) whether they perceived the central line as white or red and (ii) how visible the central line was, on a scale from 0 (not visible at all) to 3 (completely visible). Next, participants were shown 40 blocks of two identical trials each, depicting a non-offset SQM sequence with a total of six pairs of flanking lines, in which either the central line was red or the central line was not presented (i.e., black) and the first flanking line was presented in red. Participants were asked to verbally report whether they perceived the central line as not presented (i.e., black) or as red.

#### Experiment 3

Participants completed 4 blocks – 2 blocks for each of the two main conditions: V0 + AV3 (i.e., where Vernier and anti-Vernier were presented in different colors) and V0 + AV4 (where both offsets had the same color). Half of the participants started with the V0 + AV3 condition, and the other half started with the V0 + AV4 condition. Similar to experiment 1, after the first half of blocks, the block order was inverted for the second half of the experiment.

In addition to these quantitative measurements, participants were briefly presented with the stimuli and were asked two questions. First, they were asked to describe their percept of the motion stream and to match it to one of the following three descriptions: (i) a single line that is switching colors between red and white, (ii) two lines, one following the other, or (iii), something else. Second, participants were asked whether they perceived the motion trajectory as smooth or as non-smooth (i.e., as flickering). Participants were asked the same question at the beginning and at the end of the experiment, but no participant changed their reported percepts between the two sessions.

### Data analysis

In each experiment and condition, we determined the level of central Vernier dominance (or simply: “dominance”), defined as the percentage of responses matching the direction of the central Vernier offset. For example, if a central Vernier and a subsequent anti-Vernier are shown, a 100% Vernier dominance means that all responses conformed to the initial Vernier's offset direction. A 0% dominance level indicates that all responses matched the anti-Vernier's direction, whereas a 50% dominance indicates that responses matched the direction of the Vernier and that of the anti-Vernier equally often. Across the three experiments, we extracted and compared the main conditions’ dominance levels under different experimental variations.

Note that if we observe integration behavior (typically: dominance levels close to about 50%) in V + AV conditions, we will want to compare the observed dominance level with that of the AV-only condition (for additional validation, we also reported, in experiment 1, comparisons with the V-only condition). If we observe non-integration (typically: dominance levels markedly different from 50%), we will want to compare the observed dominance level with the predicted dominance level if the offsets are expected to integrate. Considering that the V and AV offset sizes are calibrated to the same value (75%), the predicted dominance level when the offsets are expected to integrate would typically be 50%. However, because certain stimulus variations (for instance, the color variations used in experiment 2) could differentially affect performance in the V and AV conditions, we determined the predicted dominance level as follows: First, we measured, for each condition, the dominance level for a sequence only depicting a Vernier (D(V)) as well as for a sequence only depicting an anti-Vernier (D(AV)). We then computed the predicted dominance level, based on Otto et al. (2009), as follows: predicted dominance = D(V) + D(AV)-50%. Thus, if the measured dominance in the V and AV conditions are 75% and 25%, respectively, the predicted dominance level would be at 50%. However, if dominance in the V condition is at 75% but dominance in the AV condition is at 35%, as opposed to 25%, the predicted dominance would be 60% (i.e., leaning more toward the initial Vernier direction). The empirically measured and theoretically predicted dominance levels can then be subsequently compared in paired-sample *t*-tests.

In addition, to directly compare the effect of percept or stimulus condition, we carried out an independent t-test to compare observed dominance levels across the two percept groups (see experiment 1) and used a repeated-measures (Bayesian) analysis of variance to compare differences between dominance level and expected integration level across the three stimulus conditions (see experiment 2).

### Power analysis

Experiments using the SQM (Otto et al., 2006) reveal large effect sizes, typically with a Cohen's d of 1.5 or above (Drissi-Daoudi et al., 2019; Drissi-Daoudi et al., 2020). Given this effect size, a power analysis revealed that a relatively small group of seven participants would be enough to achieve a power greater than 90% when using a paired-sample two-tailed *t*-test with the standard alpha level of 5%. To be on the safe side, we here recorded data from more participants. Specifically, we recruited eight participants for experiment 2, and we recruited 18 participants for experiments 1 and 3, because of the more complex analysis based on subsets of participants.

## Results

### Experiment 1

In experiment 1, we set out to examine whether, for a given participant, similarity of color, as a strong perceptual grouping cue, is linked to whether two visual features are integrated, and whether this can potentially be accounted for by a participant's perceptual report of the integrity of the motion stream. Specifically, using the SQM, we tested whether a central Vernier and a later anti-Vernier would integrate even if one or both of the offset lines “pop out” in terms of their color. To this end, we tested integration in four different conditions, as illustrated in Figure 2A: “White,” where all lines were presented in white, “Red V,” where only the central Vernier was red and all other lines white, “Red AV,” where only the anti-Vernier line was red, and “Red V + Red AV,” where both offset lines were red and all other lines white.

V and AV offsets were calibrated separately, both when presented in white and in red, to yield around 75% dominance for the central Vernier and 25 % for the anti-Vernier. When both offsets are presented in the “White” condition, they integrate as expected, and the dominance level, close to 50%, is significantly different from that of the AV-only and V-only conditions (Figure 2B; two-sided Holm paired *t*-tests: V + AV [White] vs AV-only [White]: *t*(17) = 6.650, *p*_Holm_ < 0.01, *d*′ = 1.567; V + AV [White] vs. V-only [White]: *t*(17) = 14.645, *p*_Holm_ < 0.01, *d*′ = 3.452).

In the condition “Red V,” the red central line was subjectively perceived as being separated from the stream, with low subjective ratings (1-3) on a scale from 1 (“the red element appears to be completely separated from the motion stream”) to 6 (“the red element appears to be completely part of the motion stream”). Nevertheless, Vernier and anti-Vernier integrated normally (Figure 2C), with dominance close to 50% and significantly different from when only a white anti-Vernier or a red central Vernier was presented (V + AV [Red V] vs. AV-only [White]: *t*(17) = 9.343, *p*_Holm_ < 0.01, *d*′ = 2.202; V + AV [Red V] vs. V-only [Red V]: *t*(17) = 13.496, *p*_Holm_ < 0.01, *d*′ = 3.181).

In the “Red AV” condition, 13 of the participants perceived the red line as being part of the stream (high subjective ratings between 4 and 6), whereas 5 perceived it as separate from the stream (low subjective ratings between 1 and 3). Interestingly, as is evident in Figure 2D, in both the “Red AV” and the “Red V + Red AV” condition, the two offsets integrated (i.e., dominance was close to 50%) when the red line was perceived to belong to the stream, but it integrated markedly less when it was perceived as being separate from it (i.e., dominance was below 50%). For both conditions, two-sided independent-sample *t*-tests revealed significant differences in integration behavior between individuals who rated the pop-out to be weak versus strong (V + AV dominance levels in Red AV [rating 1-3] vs. Red AV [rating 4-6]: *t*(16) = 5.326, *p*_Holm_ < 0.01, *d*′ = 2.803, and V + AV dominance levels in Red V + Red AV [rating 1-3] vs. Red V + Red AV [rating 4-6]: *t*(16) = 3.875, *p*_Holm_ < 0.01, *d*′ = 2.039).

Thus, mandatory integration in these conditions is linked to whether or not the red anti-Vernier is perceived as being part of the stream. To the contrary, the central Vernier offset information appears always to be inherited by subsequent flanking lines, regardless of whether presented in white or red.

### Experiment 2

**Figure 3.**
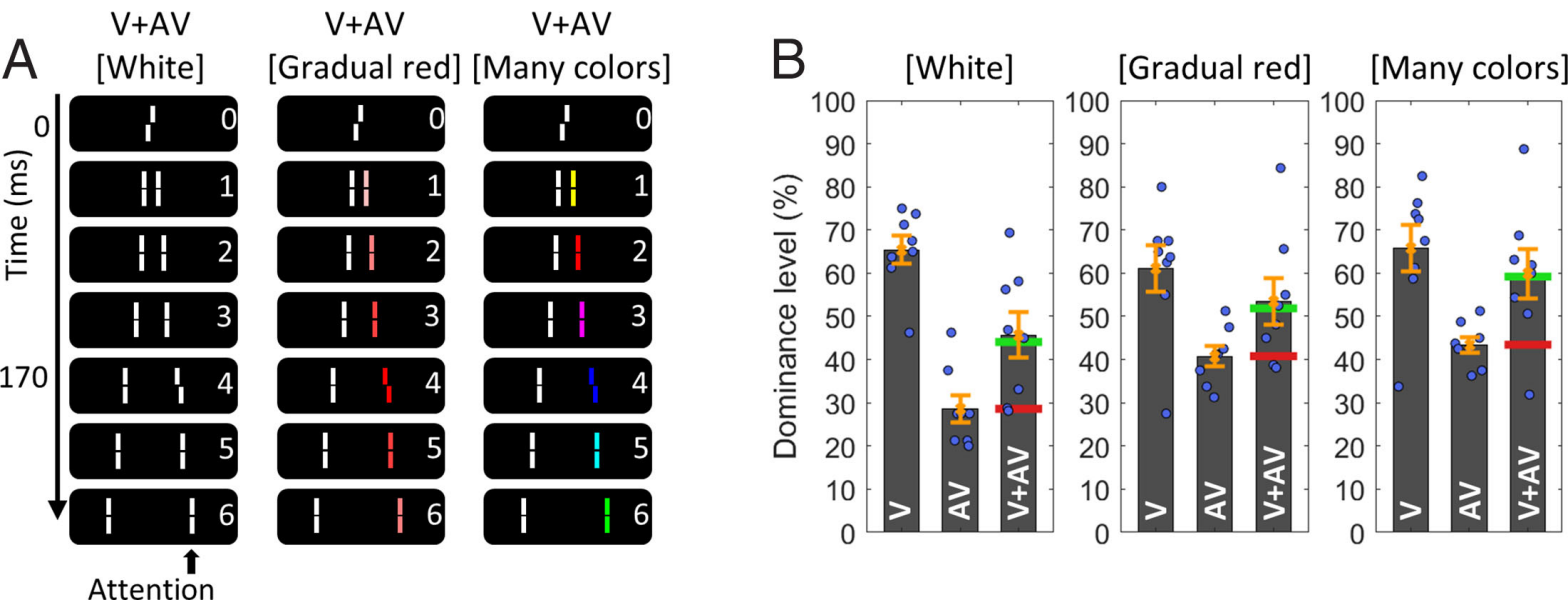
Results of experiment 2. (**A**) Visualization of our three different experimental conditions. (**B**) Central Vernier dominance levels. In all conditions, the measured V + AV dominance level is much closer to the predicted dominance level when expecting integration (green lines) than the dominance level when the AV is presented alone (red lines). Thus, in all conditions, V+AV offsets integrate despite the differences in color. Note that while the dominance observed in the V and AV conditions differs across experimental manipulations (in particular, AV dominance is closer to 50% in the “Gradual red” and “Many colors” conditions), this is accounted for in the computation of the predicted dominance level. Error bars represent the standard error.

In experiment 2, we tested for integration between a Vernier and an anti-Vernier in two additional color conditions. First, similar to the condition “Red AV” in experiment 1, we presented a white central Vernier and a red anti-Vernier at line 4, but we smoothly interpolated the color of the lines shown in between and thereafter (see Figure 3A). This was to probe whether, by reducing the “pop out” of the anti-Vernier line color, integration would be similar to that in the normal SQM sequence presented with exclusively white lines. Second, we presented a stream in which each line had a different color, to probe whether each line constituting a “pop out” in itself would create a superior pop-out and disruption of integration (or, instead, cancel out any overall pop-out, in which case normal integration would also be expected to occur).

As can be seen in Figure 3B, in both conditions, as well as in a standard “White” condition for reference, the observed V + AV dominance level is markedly close to the predicted dominance level when expecting integration (green bars), relative to the dominance level of the anti-Vernier alone (red bars). Two-tailed paired-sample *t*-tests comparing V + AV dominance and AV-dominance revealed significant differences for all three conditions (t(7) = 2.432, *p* = 0.045, *d*′ = 0.860 for the classic condition, *t*(7) = 2.554, *p* = 0.038, *d*′ = 0.903 for the gradual condition, and *t*(7) = 3.599, *p* = 0.009, *d*′ = 1.272 for the many-colors condition). In addition, a repeated-measures Bayesian analysis of variance comparing the difference between V + AV dominance levels and predicted dominance levels when expecting integration revealed moderate evidence in favor of an absence of an effect of condition (*BF*_10_ = 0.243).

Thus, as expected, with gradually appearing colors, normal integration occurs. Furthermore, when colors are changing with each successive line, there does not appear to be a very strong “pop out” effect, given that normal integration is observed as well. Considering the constant color changes in the “many colors” condition, one might have expected the latter condition to elicit a stronger pop out effect and a stronger disruption of integration, relative to what was observed in, for instance, the “Red AV” and “Red V + Red AV” conditions presented in experiment 1. That this did not happen is pointing to the possibility that a pop out may be cancelled out when several colors are changing successively. However, only a low proportion of participants reported a clear pop-out percept (five out of 18) in experiment 1, and different participants took part in both experiments. Thus it is challenging to quantify whether less of a disruption effect is elicited in the “many colors” relative to “single pop-out color” conditions. Accordingly, we can only draw very limited conclusions here.

**Figure 4.**
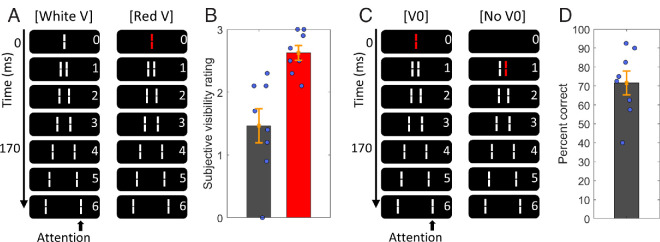
Additional results of experiment 2. (**A**) Participants were presented with an SQM sequence comprising either a white or a red central line. (**B**) Subjective reports of the visibility of the central line on a scale from 0 to 3 (0 = completely invisible, 3 = completely visible). The red central line is rated significantly more visible. (**C**) Participants were presented with an SQM sequence comprising either a red central line or a black (i.e., invisible) central line and a red flanking line. They were asked to report the perceived color of the initial line. (**D**.) Percentage of judgements that are in agreement with the factual initial line colors of the presented sequences. Error bars represent the standard error.

Further following up on the results of experiment 1, we also investigated the role of the central line's color. First, when presenting an SQM sequence with a white vs. red central line (Figure 4A), participants were able to correctly name the central Vernier color with a performance of 98.1%. Furthermore, as shown in Figure 4B, participants subjectively rated the visibility of the central line significantly higher when it was red (with a mean visibility score of 2.6 on a scale from 0 to 3) than when it was white (mean visibility score = 1.5; *t*(7) = 3.899, *p* = 0.006, *d*′ = 1.378 in two-tailed, paired-sample *t*-test).

Second, participants were presented with a stream in which either (i) the central line was red or (ii) the central line was black and the first flanking line was red (Figure 4C), and they were asked to report whether they perceived the central line as black or as red. As shown in Figure 4D, the subjective report was aligned with what was factually presented more than 70% of the time, and significantly differently so from chance level (*t*(7) = 3.451, *p* = 0.011, *d*′ = 1.220 in two-tailed, one-sample *t*-test). Note that chance level is what would be expected for a white central line (Otto et al., 2006).

Thus the visibility of the central line appears to be markedly heightened when it is presented in red rather than in white. It is important to note, however, that we cannot exclude the possibility that a higher subjective visibility score of the red central line may have, at least in part, resulted from participants exhibiting a greater certainty about the central line being perceived when it is presented in red. Furthermore, it is possible that instead of requiring a clear percept of visibility, a percept of red color in isolation (when presented at the first line) versus red being embedded into white neighboring elements (when presented at the second line, following and preceding a white line) could potentially suffice for making a judgement.

### Experiment 3

**Figure 5.**
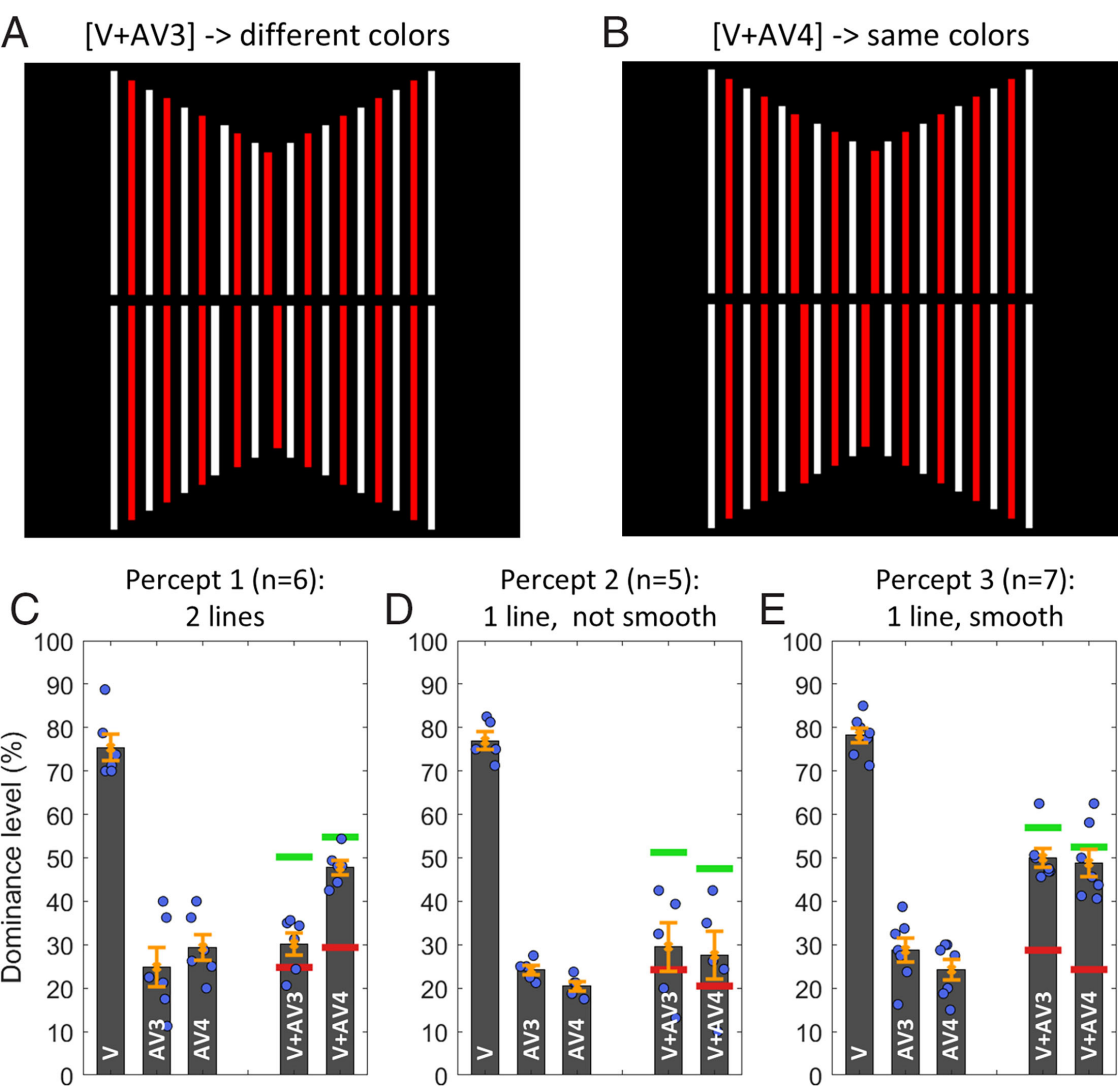
Results of experiment 3. (**A**) Visualization of the V + AV3 sequence, in which the Vernier and anti-Vernier lines were presented in different colors. (**B**) Visualization of the V+AV4 sequence, in which Vernier and anti-Vernier lines were presented in the same color. The central line is depicted in red here. In the experiment, for half of the participants, the central line was white, and for the other half red. (**C**) Central Vernier dominance levels for participants who report having perceived the motion stream as two lines, with the color of the central line following the other color and the motion being perceived as smooth. Here, integration tends to occur for the V + AV4 condition (i.e., when the colors of Vernier and anti-Vernier align) but not in the V + AV3 condition (i.e., when the two colors differ). (**D**) Central Vernier dominance levels for participants who report having perceived the motion stream as a single line that is flickering. Here, no integration appears to occur in either of the two conditions. (**E**) Central Vernier dominance levels for participants who report having perceived the motion stream as a single, smooth line. Here, integration appears to occur in both conditions. Green lines indicate the predicted dominance level when V and AV are expected to integrate; red lines represent the dominance of only the AV. Error bars represent the standard error.

In experiment 3, the SQM was presented with colors that were alternating between red and white at each successive line (Figures 5A and 5B). Across the 18 participants, three different subjective percepts were reported. First, six observers reported to have perceived two lines, moving smoothly, with the color of the central line following the other color. As can be seen in Figure 5C, for these participants, integration tends to occur when Vernier and anti-Vernier have the same color (i.e., when the anti-Vernier is presented at frame 4), with dominance levels, close to the expected integration level, being significantly different from AV-only levels (*t*(5) = 4.927, *p* = 0.004, *d*′ = 2.011 in two-tailed, paired-sample *t*-tests comparing V + AV4 dominance and AV-only dominance) but not when the colors of the two offsets differ (i.e., when the anti-Vernier is presented at frame 3), with dominance levels, close to AV-only levels, being significantly different from expected integration levels (*t*(5) = 4.030, *p* = 0.010, *d*′ = 1.645 in two-tailed, paired-sample *t*-tests comparing V + AV3 dominance and predicted integration levels). Thus, it appears that, for these participants, the red and white lines are processed as two separate objects, and mandatory integration only occurs when offsets are presented within the same extracted object.

Furthermore, five observers perceived the stimulus sequence as one line that is switching colors in a non-smooth fashion (Figure 5D). For the participants who reported this percept, no integration appears to occur in either of the two conditions, with observed V + AV dominance levels close to the AV-only levels being significantly different from the expected integration level (*t*(4) = 2.838, *p* = 0.047, *d*′ = 1.269 for V + AV3; and *t*(4) = 3.082, *p* = 0.037, *d*′ = 1.378 for V + AV4). Finally, seven observers perceived the sequence as one line that is switching its color in a smooth fashion (Figure 5E). For these participants, integration tends to occur in both conditions, with observed V + AV dominance close to the predicted integration level being significantly different from the AV-only levels (*t*(6) = 4.607, *p* = 0.004, *d*′ = 1.741 for V + AV3; and *t*(6) = 10.614, *p* < 0.001, *d*′ = 4.012 for V + AV4). Thus, similar to experiment 1, while different participants experience different percepts when varying the color dimension of the SQM, their perceptual reports align systematically with the measured dominance levels.

## Discussion

Visual features are integrated in long-lasting windows of unconscious processing that precede conscious awareness (Drissi-Daoudi et al., 2019; Herzog et al., 2016; Herzog et al., 2020; Otto et al., 2006). Here, building up on previous findings by Drissi-Daoudi et al. (2021), we showed that different subjective percepts, elicited by different perceptual groupings, are associated with different ways of integration.

Our first experiment revealed that whether a red anti-Vernier in an otherwise white stimulus stream is integrated with an initial Vernier is linked to whether the red anti-Vernier is perceived as being part of the motion stream. Specifically, we found that mandatory integration occurred for individuals who perceived the differently colored anti-Vernier line as part of the smooth motion stream, but it was markedly weaker for individuals who perceived the anti-Vernier as popping out from the stream. Along similar lines, experiment 3, in which the line colors alternated between white and red at each successive frame, revealed that integration only occurred when different offsets were perceived as being part of the same object. That is, for participants who perceived alternating red and white lines as a single smooth stream, integration was observed. Those who perceived two streams of different colors exhibited integration only if Vernier and anti-Vernier had the same color but not if they were of different colors. Finally, participants who perceived the stream as switching colors in a non-smooth fashion showed no integration in either of the conditions.

Thus, what emerges across both experiments is that participants have different percepts, and these percepts are linked to different ways of integration. These results are consistent with Drissi-Daoudi et al. (2021), who demonstrated that feature integration is conditioned on object identity and proposed that different perceptual groupings, across conditions or individuals, determine temporal integration. Individual perceptual groupings are consistent with large individual differences in vision in general (Bosten & Mollon, 2010; Cappe, Clarke, Mohr, & Herzog, 2014; Cretenoud et al., 2019; Grzeczkowski, Clarke, Francis, Mast, & Herzog, 2017; Shaqiri et al., 2019).

Our second experiment revealed that when colors are changing with each successive line, there does not seem to be a very strong “pop out” effect, as normal integration levels are observed. In light of the frequent color changes in this condition, one could have expected strong effects of perceptual pop-out and observed disruption of integration. However, as described in the Results section, because of the rather low fraction of those exhibiting a pop-out effect in experiment 1 and different participants taking place in different experiments, it is challenging to quantitatively establish whether less of a disruption effect is observed in “many colors” as compared to the “single color” conditions.

What is noteworthy about the results reported here, concerning the observed relation between subjective percepts and measured integration behavior, is that they suggest that percepts themselves may not only be the outputs of processing but may also impact processing directly. The finding that visual integration may not strictly precede the percept but that the two processes could be intertwined, is in keeping with the idea that vision cannot be both hierarchical and feedforward (Herzog & Clarke, 2014). In such hierarchical and feedforward models, progressively more complex features are proposed to be extracted along the visual hierarchy. This has the consequence that low-level processing is fully determining high-level processing but not the other way around (Herzog & Clarke, 2014). To explain the results of this study, feedback connections from object-level representations to lower visual areas, involved in Vernier integration, might be required. It is worth pointing out, however, that this does not necessitate explicit top-down Gestalt processing but could also be explained by certain local processes (Herzog, Ernst, Etzold, & Eurich, 2003). Specifically, in the shine-through effect, where a Vernier, when followed by an extended and homogeneous grating, remains visible as a “shine-through” element, even small changes to the homogeneity of the grating are known to reduce or eliminate the effect (Herzog, Fahle, & Koch, 2001). Although this may intuitively be accounted for by Gestalt principles, Herzog et al. (2003) demonstrated that a simple neural network that does not follow any such explicit Gestalt rules is nevertheless capable of reproducing these behavioral findings. Thus, it is possible that for the results reported here, local computations could suffice.

In interpreting the linkage between subjective perception and integration reported in this paper, it is critical to note, however, that a correlation between subjective reports and measured integration behavior does not necessarily imply a causal relationship of the former on the latter. Instead, other alternatives need to be considered. For instance, it is possible that instead of subjective perception determining (and, by extension, preceding) temporal integration, there could be a common source for both.

Attention may be a dimension that is especially worthy of elaboration in this context. For instance, it is possible that some individuals (covertly) attend to the presented stimuli differently because the color change is more meaningful for them. This difference in attention could potentially account for some of the inter-individual differences at the level of both subjective perception and observed integration behavior. Moreover, it is conceivable that disrupted integration could be a consequence of a “pop out” only if it occurs at the level of a single element and only if such element is attended. It would be interesting for future work to examine what differentiates individuals who report pop outs from those who do not. Such investigations could include, for instance, tests of color sensitivity, attention, and perception of smoothness of individual lines even in normal SQM streams, and could help differentiate between different explanatory accounts of the findings reported here.

Relatedly, an open question concerns the effect of the unattended stream of the SQM. That is, while the attended stream was modified in all three experiments reported here, the unattended stream (with the exception of the central Vernier) was always presented in white for experiments 1 and 2. Even if unattended, this may contribute to the percept of continuity, and there could be inter-individual differences of such percept. Future studies with similarly modified unattended streams could help test such hypotheses.

Relatedly, it is worth noting that we do not yet have a clear explanation for why full integration occurred when a red initial Vernier was displayed in an otherwise white motion stream in our first experiment. This is puzzling considering that the red Vernier was subjectively reported to pop out of the stream and considering that experiment 2 confirmed that red central lines are indeed visible. One possibility emerges when considering predictive processes. Specifically, an anti-Vernier that is presented in red differs from the previous lines both in terms of its color and its spatial offset information. Considering this prominent difference across two important stimulus dimensions, it is likely that a disruption in the stimulus regularity is detected. In contrast, if the red Vernier is presented at the very beginning of the stream, no such expectation would be built up and no comparable “prediction error” would be elicited.

The finding reported here may further complement past studies in the context of object-substitution masking (Enns & Di Lollo, 1997). This describes the phenomenon that whether or not a briefly-presented target is perceived depends on the presence of a second, spatially non-overlapping mask that appears at the same time but disappears slightly later. Past work has shown that when the target and mask differ in terms of stimulus properties such as color, the masking effect is reduced (Moore & Lleras, 2005). Such findings have been taken as evidence for an “object updating” rather than an “object substitution” framework, suggesting that object representations (of both the target and mask) are continuously updated (see Goodhew, 2017 for review). The experiments conducted here, carried out with a spatiotemporally more extended paradigm, complement such studies and reveal important differences between participants, as well as between colored Verniers appearing at the beginning or in the middle of the motion stream.

Although the main objective of this article was to examine the relationship between subjective percepts and measured integration behavior, it is also worth noting that in the first experiment, relatively few participants exhibited pop-out percepts and an according disruption of temporal integration (with only five out of 18 participants reporting a pop-out rating of 3 or less, on a scale from 1-6). This supports the general notion that integration in the SQM is typically fairly stable (Drissi-Daoudi et al., 2021; Vogelsang et al., 2024).

Finally, it is worth noting that while the SQM has very large effect sizes, and while we used many more participants than would be required in typical SQM experiments, due to the complex nature of the experiments and subset-based analyses reported here, the sample size used may constitute a limitation. In addition to replicating the results presented here, future work could include additional perceptual reports.

Notwithstanding some open questions, our results strengthen the case for complex and participant-specific perceptual grouping mechanisms being linked to temporal integration behavior. Perception furthermore emerges not only as the outcome of temporal integration but potentially also as part of it. Finally, since we are not aware of these processes, our results provide further evidence for long-lasting unconscious processing preceding discrete conscious perception, as proposed in Herzog et al. (2016); Herzog et al. (2020) (though see Fekete, Van de Cruys, Ekroll, & van Leeuwen [2018] and Doerig, Scharnowski, & Herzog [2019] for discussion).
